# Absolute position versus relative position in embryo transfer: a randomized controlled trial

**DOI:** 10.1186/s12958-015-0072-6

**Published:** 2015-07-29

**Authors:** Hwang Kwon, Dong-Hee Choi, Eun-Kyung Kim

**Affiliations:** Department of Obstetrics and Gynecology, CHA Fertility Center of Bundang CHA General Hospital, CHA University, Seongnam, Korea; CHA Fertility Center of Bundang CHA General Hospital, CHA University, Seongnam, Korea; College of Medicine, CHA University and CHA Fertility Center of Bundang CHA General Hospital, 351 Yatap-dong, Bundang-gu, Seongnam, Gyeonggi-do 463-712 Korea

**Keywords:** Site of embryo transfer, Absolute position, Relative position, fundal endometrium, Endometrial cavity length

## Abstract

**Background:**

Meta-analysis revealed that embryo placement 20 mm from the fundal endometrial surface resulted in higher pregnancy rate, ongoing pregnancy rate, and live birth rate compared with placement 10 mm from the fundal endometrial surface. Pregnancy and implantation rates according to relative position were higher when the catheter tip was positioned close to the middle of the endometrial cavity. The aim of the current study is to evaluate differences in implantation and pregnancy rates if the site of embryo transfer is 2 cm distance from the fundal endometrium (DFE) compared to the midpoint of the endometrial cavity length (ECL).

**Methods:**

Patients were randomized to one of two groups: in group A (n = 98, 98 IVF-ET cycles), the embryo transfer catheter tip was positioned 2 cm DFE, while that in group B (n = 97, 97 IVF-ET cycles) was positioned at the midpoint of the ECL. We compared pregnancy outcomes of implantation rate, chemical pregnancy rate, clinical pregnancy rate, ongoing pregnancy rate, ectopic pregnancy rate, and miscarriage rate in the two groups.

**Results:**

Analysis of implantation rate (19.5 ± 27.7 vs. 21.7 ± 32.6; p = 0.6), chemical pregnancy rate (51 % vs. 50.5 %; p = 0.94), clinical pregnancy rate (35.7 % vs. 38.1 %; p = 0.73), ongoing pregnancy rate (31.6 % vs. 30.9 %; p = 0.92), ectopic pregnancy rate (8.6 % vs. 2.7 %; p = 0.35), and miscarriage rate (11.4 % vs. 16.2 %; 0.74) revealed comparable results for both groups.

**Conclusions:**

Implantation and pregnancy rates were not influenced by the site of the ET catheter tip being 2 cm DFE compared to at the midpoint of the ECL.

**Trial Registration:**

ISRCTN: ISRCTN15972342

## Background

Embryo transfer is one of the most important factors affecting the rate of successful pregnancy in IVF-ET. Variables in ET such as removal of cervical mucus [1, 2], sonoguidance [3–6], catheter type [7–13], catheter loading technique [14–16], presence of blood on the catheter tip [17], bacterial contamination [18, 19], and site of embryo deposition [20–22] are all determinants of a successful pregnancy.

There has been debate regarding which area within the endometrial cavity is ideal for embryo placement in order to obtain the highest pregnancy rate. Various studies regarding the relationship between embryo transfer site and pregnancy outcome have been divided analysis into two categories: absolute position according to distance from the fundal endometrium (DFE) and relative position according to endometrial cavity length (ECL). It is not easy to determine whether the embryo deposition site should be chosen based on absolute position from the fundal endometrium or by relative position according to endometrial cavity length. In a previous randomized controlled trial (RCT) on absolute position, pregnancy rate was significantly higher when the embryo was deposited 20 mm caudal to the fundus compared to when it was deposited 10 mm caudal to the fundus (60 % vs. 39.3 %) [20]. A meta-analysis found that embryo placement 20 mm from the fundal endometrial surface resulted in higher pregnancy rate, ongoing pregnancy rate, and live birth rate compared with placement 10 mm from the fundal endometrial surface [23]. One research group reported that pregnancy and implantation rates did not vary according to the distance between the catheter tip and fundal endometrium (group 1: 10–15 mm, group 2: 16–20 mm, and group 3: ≥21 mm), but that pregnancy and implantation rates according to the relative position of the catheter tip in endometrial cavity were higher when the catheter tip was positioned close to the middle of the endometrial cavity (group 1: upper 40 % ECL, group 2: upper 40 % to midpoint ECL, group 3: from the midpoint to lower 40 % ECL, and group 4: lower 40 % ECL) [22].

Although discrepancies exist regarding pregnancy rates according to the absolute or relative position of the catheter tip, a 2-cm DFE was considered the reference point for absolute position because that point was associated with a higher pregnancy rate in a meta-analysis [23]. As observed pregnancy and implantation rates were higher in the midpoint ECL in a RCT evaluating relative position, midpoint ECL was considered the reference point for relative position. The aim of this current prospective RCT is to compare the differences in implantation and pregnancy rates at an absolute position 2 cm from the fundal endometrium and a relative position at the midpoint of the endometrial cavity.

## Methods

This randomized controlled trial was approved by the Institutional Review Board at our hospital. A total of 197 patients were enrolled in the IVF-ET (ICSI) program at the CHA Fertility Center (CHA University, Seongnam, Korea) between July 2012 and December 2014. All patients provided written informed consent. The study was registered under ISRCTN registry number (ISRCTN 15972342).

### Sample size and randomization

The null hypothesis is that the rates for groups A and B are equal. The sample size was calculated to prevent type II errors. In previous articles, pregnancy rates at 2 cm distance from fundal endometrium [20] and the central area of the endometrial cavity [22] were 60 % and 36.7 %, respectively. Based on these differences, 84 cases in each group would be needed with a two-sided alpha level of 0.05 and a beta level of 0.20 (80 % power). This sample size calculation was done so that the null hypothesis was not accepted with a probability of 80 %. Thus, the target number of subjects for each group was 100 (a total of 200) to allow dropouts. Randomization was performed using a computer-generated random number list in blocks of four. An allocation list was sealed in an envelope and the physician transferring embryos opened the envelope just before ET.

### Study design

**Fig. 1:**
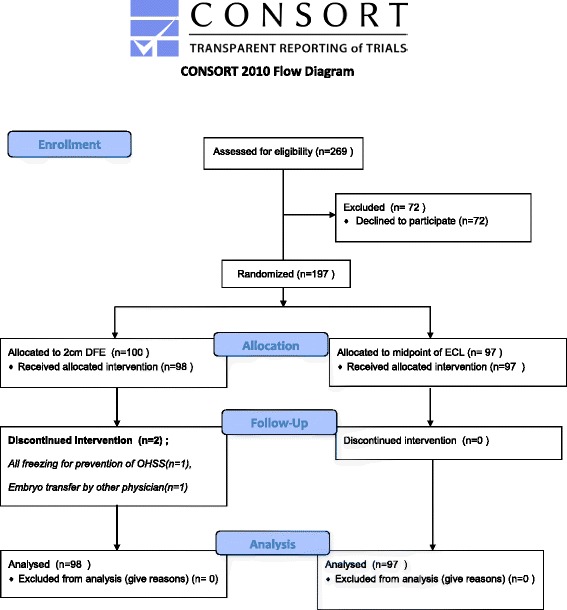
Consolidated Standards of Reporting Trials (CONSORT) diagram showing flow of patients through the study. DFE, distance from fudal endometrium. ECL, endometrial cavity length. OHSS, ovarian hyperstimulation syndrome

197 patients were randomized to one of two groups: group A or group B. In group A (n = 100), the embryo transfer catheter tip was positioned 2 cm from the fundal endometrium. In group B (n = 97), the embryo transfer catheter tip was positioned at the midpoint of the endometrial cavity length, between the internal os of the uterine cervix and the fundal endometrium. The current study included only fresh embryo transfer cycles. Two patients were excluded from group A, including one patient in which all embryos were frozen for prevention of OHSS and the other patient in which embryos were not transferred by the same physician due to the absence of a physician (Fig. 1).

### Stimulation protocol and oocyte retrieval

All patients were pretreated with GnRH agonist (Lorelin®; Dongkook, Korea) 0.5 mg/day from 10 days prior to the start of menstruation until day 2 of the next menstrual cycle for suppression of the pituitary gland. The patients’ ovaries were stimulated with a combination of recombinant follicle-stimulating hormone (FSH, subcutaneous) and human menopausal gonadotropin (hMG, intramuscular) from day 3 of the menstrual cycle until one day before hCG administration. hCG (Ovidrel®; Merck-Serono, Italy) was administered when at least two follicles with an average diameter ≥18 mm were observed on ultrasonography. Oocytes were retrieved 35 hours after hCG injection and were subsequently fertilized by IVF or ICSI. On day 2 or 3, all embryos were evaluated for cell number and morphology. Each embryo transferred was evaluated for blastomere size and fragmentation. Embryos were graded as follows. Those with equal blastomere size and no fragmentation were considered Grade 1; those with blastomeres of equal size with slight fragmentation (<20 %) were Grade 2; those with blastomeres of unequal size but no fragmentation were Grade 3; those with blastomeres of equal or unequal size and moderate fragmentation (20 %–50 %) were Grade 4; and those with unrecognizable blastomeres and severe fragmentation (>50 %) were Grade 5 [24]. The embryos were transferred on postretrieval day 2 or 3.

### Embryo transfer

After bladder filling, patients were placed in the lithotomy position, and the cervix was visualized using a speculum. The vagina and exocervix were cleaned with sterile gauze, and endocervical mucus was removed with a cotton swab. We measured the distance between the internal os of the cervix and the fundal endometrium using 5 MHz transabdominal ultrasonography (Aloka SSD-4000; Hitachi Aloka Medical Ltd., Japan) in both groups of patients. This distance was measured only once and was considered the endometrial cavity length (ECL). The ECL and the midpoint of the ECL were recorded. A Sydney-Cook catheter external guide was inserted into the endometrial cavity through the endocervical canal. We measured the distance from the tip of the external guide to the fundal endometrium only once and estimated the position of the internal catheter tip loaded with the embryos. After the internal catheter tip passed through the external guide and was positioned 2 cm from the fundal endometrium (DFE) in group A and at the midpoint of the ECL in group B, the embryos were expelled. The catheter was immediately removed and examined under a stereomicroscope to determine whether the embryos remained in the catheter tip. It was recommended that patients remain supine in bed for 2 hours after emptying their bladder [25]. All embryo transfers were performed by the same physician.

Micronized progesterone (Utrogestan® 200 mg Vaginal Capsule; Capsugel, France) was inserted into the vagina three times per day beginning on the day of oocyte retrieval for luteal support. Pregnancy was determined by serum β hCG level 13 days after embryo transfer.

### Outcome measures

To determine the difference in general characteristics of patients, the following factors were compared in groups A and B: age, etiology of infertility, body mass index (BMI), AMH, antral follicle count (AFC), and duration of infertility. Also, for comparison of ovarian stimulation, oocyte retrieval, and IVF outcome in groups A and B, the following factors were recorded: E_2_ and endometrial thickness on the day of hCG administration, number of oocytes retrieved, number of oocytes in metaphase II, fertilization rate, fertilization method, number of embryos transferred, good quality embryos (%, Grade 1 or 2 embryos according to the embryo grading system described above) [24], and endometrial cavity length. We analyzed pregnancy outcomes including implantation rate, chemical pregnancy rate, clinical pregnancy rate, ongoing pregnancy rate, ectopic pregnancy rate, and miscarriage rate. The implantation rate was defined as the number of gestational sacs divided by the number of embryos transferred. Clinical pregnancy was defined as the presence of a gestational sac with fetal heart activity. Ongoing pregnancy was defined as the presence of at least one fetal heart pulsation on ultrasound beyond 12 weeks.

### Statistics

SPSS version 22 was used for statistical analysis. Data are expressed as mean ± SD or percentage, as appropriate. Student’s *t*-test, *χ*^2^ test, and Fisher’s exact test were used to determine statistical significance. A *p*-value <0.05 was considered statistically significant.

## Results

The groups were not significantly different with respect to age, etiology of infertility, BMI, AMH, or AFC. However, there was a significant difference in duration of infertility (p = 0.025): 3.9 ± 2.1 years in group A compared with 4.8 ± 3.1 years in group B (Table 1). There were no significant differences between groups A and B with regard to E_2_ and endometrial thickness on the day of hCG administration, number of oocytes retrieved, number of oocytes in metaphase II, fertilization rate, fertilization method, number of embryos transferred, or percentage of good quality embryos. The two groups were also not significantly different with regard to endometrial cavity length (4.8 ± 0.7 vs. 4.8 ± 0.8; p = 0.94) or the difference between midpoint and 2-cm DFE (0.4 ± 0.3 vs. 0.4 ± 0.4; p = 0.98) (Table 2). Mean DFE was 2 cm in group A and 2.38 ± 0.4 in group B. Analysis of implantation rate (19.5 ± 27.7 vs. 21.7 ± 32.6; p = 0.6), chemical pregnancy rate (51 % vs. 50.5 %; p = 0.94), clinical pregnancy rate (35.7 % vs. 38.1 %; p = 0.73), ongoing pregnancy rate (31.6 % vs. 30.9 %; p = 0.92), ectopic pregnancy rate (8.6 % vs. 2.7 %; p = 0.35), and miscarriage rate (11.4 % vs. 16.2 %; 0.74) revealed comparable results for both groups (Table 3).

**Table 1:** General characteristics of all patients studied

	Group A:DFE 2 cm	Group B: midpoint of ECL	p value
Patients (n)	98	97	
cycles (n)	98	97	
Age (years) (±SD)	34.1 ± 3.8	34.1 ± 3.4	0.87
Etiology			0.55
Tubal factor	19	21	
Male factor	8	12	
POR	7	4	
Hypo-Hypo	1	0	
POR + tubal	0	1	
uterine	0	1	
Unexplained	63	58	
BMI (Kg/m2) (±SD)	21 ± 2.4	21.4 ± 3.2	0.33
AMH (ng/ml) (±SD)	3.5 ± 2.9	3.6 ± 3.2	0.67
AFC (±SD)	20.6 ± 8.6	21.5 ± 8.0	0.44
Duration of infertility (yrs,) (±SD)	3.9 ± 2.1	4.8 ± 3.1	0.025

DFE = distance from fundal endometrium, ECL = endometrial cavity length

POR = poor ovarian response, Hypo-Hypo = Hypogonadotropic hypogonadism

AFC = antral follicle count

**Table 2:** Ovarian stimulation, oocyte retrieval, and IVF outcome of all patients studied

	Group A: DFE 2 cm	Group B: midpoint of ECL	p value
E_2_ on hCG day (pg/mL) (±SD)	2740.9 ± 1413.3	2787.7 ± 1544.4	0.83
Endometrial thickness on hCG day (mm) (±SD)	10.4 ± 2.2	10.1 ± 2.2	0.3
No. of oocyte retrieval (±SD)	12.5 ± 6.5	13.4 ± 6.8	0.32
No. of oocytes in metaphase II (±SD)	7.3 ± 4.9	7.9 ± 5.0	0.41
Fertilization rate (%) (±SD)	68.6 ± 18.7	68.3 ± 17.4	0.92
Fertilization method			0.61
Conventional IVF	9	8	
ICSI	51	44	
Split IVF-ICSI	38	44	
No. of embyos transferred (±SD)	2.8 ± 0.8	2.8 ± 0.7	0.78
Good quality embyos (%) (±SD)	70.7 ± 34.8	76.7 ± 29.7	0.19
ECL (cm) (±SD)	4.8 ± 0.7	4.8 ± 0.8	0.94
Difference between midpoint and DFE2cm (±SD)	0.4 ± 0.3	0.4 ± 0.4	0.98

DFE = distance from fundal endometrium, ECL = endometrial cavity length

**Table 3:** Implantation rates, pregnancy rates, and outcome at gestation of all patients studied

	Group A: DFE2cm	Group B: midpoint of ECL	p value
Implantation rates (%)	19.5 ± 27.7	21.7 ± 32.6	0.6
Chemical pregnancy rates	51.0 %(50/98)	50.5 %(49/97)	0.94
Clinical pregnancy rate	35.7 %(35/98)	38.1 %(37/97)	0.73
Ongoing pregnancy rates	31.6 %(31/98)	30.9 %(30/97)	0.92
Ectopic pregnancy	8.6 %(3/35)	2.7 %(1/37)	0.35
Miscarriage rate	11.4 %(4/35)	16.2 %(6/37)	0.74
Mole	0 %	2.8 %(1/37)	1

DFE = distance from fundal endometrium, ECL = endometrial cavity length

## Discussion

This randomized controlled study demonstrated that implantation and pregnancy rates of patients in whom the embryo transfer catheter tip was positioned 2 cm from the fundal endometrium were comparable to those of patients in whom the embryo transfer catheter tip was positioned at the midpoint of the endometrial cavity.

One study group observed position of 3 ~ 6 mm early gestational sac (G sac) in the endometrial cavity after spontaneous pregnancy using three-dimensional ultrasound. When the uterine cavity was divided into three portions (upper, middle, and lower), most G sacs were found in the upper region (89.1 %) [26]. In another study, the location of the G sac was determined via three-dimensional ultrasound after embryo transfer in IVF-ET and 84.4 % of the G sacs were found in the fundal area where embryos were initially transferred. These results emphasize the importance of embryo transfer [27].

Endometrial blood flow is especially rich around the uterotubal junction outside of the fundal areas. When the G sac is observed daily, it develops near the uterotubal junction and fundus, gradually moving towards the central portion of uterine body [28]. Embryos are apt to implant at a site where endometrial blood flow is rich. Although little research has been done on factors related pregnancy and implantation rates according to deposition of embryos during transfer, the difference in blood flow in the endometrial cavity may play a role.

Many initial reports stated that a site near the fundus is optimal for embryo transfer [29, 30]. However, subsequent studies reported that fundal irritation due to high replacement cause fundal contraction and adversely affect pregnancy rates [31–33].

One retrospective study suggested that, for every additional millimeter the embryos are deposited away from the fundus, the odds of clinical pregnancy increase by 11 % [34]. Another retrospective study reported a higher pregnancy rate with embryo deposition >10 mm to <20 mm from the fundus [35]. In a previous RCT, higher pregnancy and implantation rates were achieved when the tip was placed between 5 and 15 mm DFE compared with >15 mm DFE [36]. Another RCT reported that an embryo transfer catheter tip positioned between 10 and 15 mm from the fundus achieved higher clinical pregnancy rate than an embryo catheter tip positioned ≤10 mm from the fundus [37]. When patients were classified into three groups including DFE of 10 mm, 15 mm, and 20 mm in an earlier RCT, DFE of 20 mm showed a significantly higher clinical pregnancy rate than DFE of 10 mm (60 % vs. 39.3 %) [20].

Consistent results were not observed in the above RCT studies with respect to absolute position from the fundal endometrium. However, a meta-analysis of three articles [20, 21, 38] using the Mantel − Haenszel method and utilizing the fixed-effects model revealed that clinical pregnancy rate, ongoing pregnancy rate, and live birth rate were significantly higher for a 20 mm versus 10 mm distance from the fundal endometrium [23]. Based on this meta-analysis, a 2-cm DFE was considered the optimal site of absolute position in the current study.

One prospective cohort study divided patients into two groups: a fundal group (embryos were deposited within 0.5-1.0 cm of the uterine fundus) and a lower-to-middle group (embryos were deposited at the central portion of the ECL to the lower third of the ECL). Comparison of these two groups revealed that clinical pregnancy rate, implantation rate, and birth rate were significantly higher in the lower-to-middle segment ET group compared with the fundal ET group [39]. Another RCT showed that placing embryos in the upper half of the uterine cavity did not improve pregnancy rate compared with that of embryos placed in the lower half [21]. These research group randomized patients into three groups according to the distance between the fundal endometrial surface and the catheter tip (group 1: 10–15 mm, group 2: 16–20 mm, group 3: ≥21 mm) and compared the pregnancy and implantation rates between groups. They also randomly assigned patients to four groups according to the relative position in the endometrial cavity (group 1: <40 %, group 2: 41-50 %, group 3: 51-60 %, group 4: ≥61 %) and compared the pregnancy and implantation rates between groups. Pregnancy and implantation rates according to absolute position were not significantly different, but pregnancy and implantation rates according to relative position were higher when the catheter tip was positioned close to the middle of the endometrial cavity [22]. When we analyzed these three studies with respect to relative position of embryo transfer, the optimal site for relative positioning of the embryo transfer was the midpoint of the ECL.

The current study was designed to compare absolute position (2 cm DFE) with relative position (midpoint of the ECL) of embryo transfer. In comparing general characteristics of all patients studied, the duration of infertility in group B (midpoint of the ECL) was significantly higher than that of group A (2 cm DFE). However, this difference in duration of infertility did not seem to correlate with dissimilarity in general characteristics, since there were no differences between groups in age, BMI, etiology of infertility, or ovarian reserve indicators (AMH, AFC). There were also no differences in implantation, clinical pregnancy, or ongoing pregnancy rates between the two groups. The mean difference between the midpoint (group A) and 2-cm DFE (group B) was only 4 mm. In most other reports, embryo transfer depth was classified in 5-mm increments from the fundal endometrium [22, 35, 37]. Thus, a difference of less than 5 mm is not considered to affect pregnancy outcomes. Overall, the minimal difference in DFE between groups A and B resulted in no differences in implantation, clinical pregnancy, or ongoing pregnancy rate.

Therefore, patients with a greater than 0.5 cm difference between the midpoint of the ECL and 2 cm DFE (in other words, an ECL ≥5 cm or ≤3 cm) were classified separately. However, there were no patients with an ECL ≤ 3 cm. A total of 37 women in group A and 34 women in group B had an ECL ≥5 cm. The mean ECL of patients with an ECL ≥5 cm was 5.5 ± 0.5 cm in group A and 5.6 ± 0.6 cm in group B (p = 0.41). The ongoing pregnancy rate for group A (DFE 2 cm) was significantly higher than that of group B (midpoint of ECL) (43.2 % vs. 20.6 %; p = 0.002) in women with an ECL ≥5 cm. In cases in which the ECL was longer than 5 cm, our data suggest that positioning the embryo transfer catheter tip more than 2.5 cm from the fundal endometrium compared with 2 cm from the fundal endometrium worsens pregnancy outcome.

Although embryo deposition at a relative 50 % ECL may be the best approach in most patients, optimal pregnancy rates in patients with an ECL ≥5 cm are obtained when embryos are placed at a 2-cm DFE.

## Conclusions

In conclusion, implantation and pregnancy rates were not influenced by the site of the ET catheter tip being 2 cm DFE compared to at the midpoint of the ECL. Since the midpoint ECL in most patients of the relative position group is near the 2-cm DFE, there was no difference in pregnancy outcomes between groups. However, if the ECL is longer than 5 cm, positioning at the midpoint of the ECL was not advantageous. Although the number of patients with an ECL ≥5 cm was small, placing the embryo catheter tip more than 2.5 cm from the fundal endometrium decreased pregnancy rates in patients with an ECL ≥5 cm. Further large-scale studies are needed in patients with an ECL ≥5 cm.
